# Tolerability of nintedanib in the elderly with idiopathic pulmonary fibrosis: A single-center retrospective study

**DOI:** 10.1371/journal.pone.0262795

**Published:** 2022-02-03

**Authors:** Masamichi Komatsu, Hiroshi Yamamoto, Takashi Ichiyama, Satoshi Kawakami, Takeshi Uehara, Yumi Yoshikawa, Yoshiaki Kitaguchi, Atsuhito Ushiki, Masanori Yasuo, Masayuki Hanaoka

**Affiliations:** 1 First Department of Internal Medicine, Shinshu University School of Medicine, Matsumoto, Japan; 2 Department of Radiology, Shinshu University School of Medicine, Matsumoto, Japan; 3 Department of Laboratory Medicine, Shinshu University School of Medicine, Matsumoto, Japan; 4 Department of Pharmacy, Shinshu University School of Medicine, Matsumoto, Japan; University of Milano Biococca, ITALY

## Abstract

Idiopathic pulmonary fibrosis (IPF), a fibrosing interstitial lung disease, predominantly affects the elderly and is associated with a high mortality risk. Nintedanib, a tyrosine kinase inhibitor, significantly reduces IPF progression. However, data on the tolerability and efficacy of nintedanib in the elderly with IPF are limited. Therefore, this study aimed to examine the tolerability and efficacy of nintedanib in the elderly with IPF in a real-world setting. Medical records of 19 elderly IPF patients (≥ 75 years) and 46 non-elderly IPF patients (< 75 years) newly administered nintedanib were retrospectively analyzed. We compared the forced vital capacity (FVC) level, incidence and severity of adverse events, and continuation rates of nintedanib between the two groups. FVC and percent predicted diffusing capacity of the lung for carbon monoxide (DLco) were lower in the elderly IPF group at baseline. Although the elderly IPF patients had a significantly higher incidence of adverse events, such as diarrhea, nausea, and elevation of hepatic enzymes, the rate of discontinuation of nintedanib owing to adverse events was not different between the groups. The continuation rates of nintedanib treatment at 6 months and 1 year in the elderly IPF group were equivalent. Furthermore, there was a similar trend in the reduction of the annual FVC decline after nintedanib initiation between the groups. Our study demonstrated that nintedanib was tolerable in both the IPF patient groups in a real-world setting. Proper management of adverse events in the elderly with IPF would lead to a better clinical outcome.

## 1. Introduction

Idiopathic pulmonary fibrosis (IPF) is the most common form of idiopathic interstitial pneumonias [1, 2]. IPF is a chronic and progressive fibrosing interstitial lung disease (ILD) of unknown etiology [1, 3]. The prognosis of IPF is as poor as many types of cancers, with a median survival of 2–3.5 years [4–6]. Because the clinical course is heterogeneous, the prognosis of IPF is difficult to predict [1, 7, 8]. Mortality in the case of IPF has been associated with the occurrence of dyspnea, poor pulmonary function, poor exercise capacity, and extent of lung fibrosis on high-resolution computed tomography (HRCT) [1]. Recently, a multidimensional index and staging system using clinical (e.g., gender and age) and physiological (e.g., forced vital capacity [FVC] and diffusing capacity of the lung for carbon monoxide [DLco]) variables, GAP, has been reported [9].

Nintedanib is a triple kinase inhibitor targeting the vascular endothelial growth factor, fibroblast growth factor, and platelet-derived growth factor [10]. The phase III study [11] demonstrated that nintedanib significantly reduced the decline of FVC in patients with IPF. In addition, the INPULSIS-ON trial [12] revealed the long-term benefit of slowing disease progression. The European IPF registry [13] demonstrated that patients treated with antifibrotic agents such as nintedanib and pirfenidone have a statistically prolonged survival rate when compared with those not on antifibrotic therapy. Nintedanib has been recommended for the treatment of IPF in the international clinical practice guideline [14]. Antifibrotics is recommended for use even in early-stage IPF patients. In addition, a recently published phase III study demonstrated the efficacy and safety of nintedanib in patients with systemic sclerosis-associated ILD (SSc-ILD) [15] and those with non-IPF progressive fibrosing ILD (PF-ILD) [16]. From these studies, it is expected that the frequency of use of nintedanib will increase.

The management of nintedanib-associated adverse events is essential during chronic therapy. The most frequent adverse events associated with nintedanib are gastrointestinal symptoms, such as diarrhea, nausea, and elevation of hepatic enzymes [11, 15, 16]. In the INPULSIS trials [11], although approximately 60% of patients suffered from diarrhea, less than 5% of patients discontinued nintedanib treatment.

IPF occurs more frequently in elderly patients, and the incidence increases with age [1, 17]. The mean age of onset is 70 (standard deviation [SD]: 9.0) years in Japan [6]. However, the available data on the tolerability and efficacy of nintedanib in elderly patients in the real-world are limited [18]. The mean age of patients who were treated with nintedanib in a real-world setting was 66.6 (SD: 8.1) years in the INPULSIS trials [11], 71 (SD: 8) years in the USA [19], and 71.8 (SD: 7.5) years in Greece [20]. In this study, we retrospectively analyzed the tolerability and efficacy of nintedanib in elderly patients aged ≥ 75 years in a real-world setting.

## 2. Methods

### 2.1 Study subjects

This study was approved by the Ethics Committee of the Shinshu University School of Medicine (Approval Number 4591) and was performed in accordance with the Declaration of Helsinki and subsequent amendments. The institutional Review Board of Shinshu University School of Medicine waived the need for informed consent owing to its retrospective design; the opportunity to opt-out was provided on the website. However, those who did not provide consent were excluded from this study.

This was a retrospective, single-center study. We reviewed medical records of patients with IPF newly administered nintedanib at a dose of 150 mg twice daily at the Shinshu University Hospital between November 2015 and March 2020. In this study, “elderly IPF” is defined as IPF in patients aged ≥ 75 years and “non-elderly IPF” as IPF in patients aged < 75 years. Sixty-eight patient records were eligible for review. Three were excluded from this study because they were being treated for acute exacerbation. Other causes of ILD, such as chronic hypersensitivity pneumonia and connective tissue diseases, were excluded with multidisciplinary discussion (MDD). Therefore, 65 (19 elderly and 46 non-elderly) IPF patients were enrolled in this study.

The diagnosis of IPF was made in accordance with the 2018 Clinical Practice Guideline Summary for Clinicians: Diagnosis of Idiopathic Pulmonary Fibrosis [3]. The decision to initiate nintedanib treatment was made by the physician after MDD. Most patients were treated with nintedanib because of FVC deterioration. Some patients were treated with the drug because of symptoms or the extension of lung fibrosis on HRCT without confirming FVC decline.

### 2.2 Clinical, physiological, and radiological findings

The baseline clinical, physiological, and radiological findings were obtained from the medical records, and the baseline GAP stage [9] was calculated. The pulmonary functions at 1 year (± 6 months) before initiation, baseline, and 1 year (± 6 months) post-initiation were compared in evaluable cases. The incidence of acute exacerbations and lung cancer were obtained. HRCT chest images obtained within 3 months of nintedanib initiation were analyzed by a radiologist (S.K.), and the HRCT pattern was determined according to the 2018 guidelines of IPF [3]: usual interstitial pneumonia (UIP), probable UIP, indeterminate for UIP, or alternative diagnosis.

### 2.3 Adverse events and tolerability

Adverse events associated with nintedanib were evaluated for severity according to the common terminology criteria for adverse events version 5.0 (CTCAE ver. 5.0). The incidence of adverse events leading to at least one dose reduction or treatment discontinuation as well as any therapeutic interventions applied to manage them were analyzed. We further analyzed the duration of the first dose reduction and treatment discontinuation as well as why the patient discontinued the nintedanib treatment.

### 2.4 Statistical analysis

Continuous data were presented as mean (SD) or median (range) and categorical data as the number in each group. While the Mann-Whitney *U* test was used to compare continuous variables between the two groups, the χ^2^ test or the Fisher’s exact test was used to compare categorical variables. The Kruskal-Wallis test was used to compare the variables in three or more groups. The paired Wilcoxon-rank test was used to compare annual FVC decline in groups with available data. The overall survival (OS), median, and 95% confidence intervals (CIs) were determined using the Kaplan–Meier method, whereas the differences between groups were compared using the log-rank test. Multivariate analysis was performed using logistic regression. The statistical analyses were performed using StatFlex^®^ Version 7.0 (Artech, Osaka, Japan). The statistical significance was established at *p* values of < 0.05.

## 3. Results

### 3.1 Patient characteristics at baseline

The baseline characteristics obtained are shown in Table 1. Five patients were diagnosed with IPF using surgical lung biopsy samples, and the other patients were diagnosed with clinical IPF after MDD. There were significant differences between the elderly IPF and non-elderly IPF groups in terms of mean age (78.2 ± 2.1 vs. 66.5 ± 7.4 years; *p* < 0.001) and body mass index (BMI) (22.6 ± 3.9 vs. 24.6 ± 3.4 kg/m^2^; *p* = 0.040), respectively. For laboratory data, there were significant differences in levels of albumin (3.75 ± 0.54 vs. 4.04 ± 0.35 g/dL; *p* = 0.013) and C-reactive protein (CRP) (1.48 ± 2.98 vs. 0.49 ± 0.90 mg/dL; *p* = 0.040) between the elderly and non-elderly groups, respectively. One patient (5.3%) and five patients (13.0%) in the elderly IPF and non-elderly IPF groups, respectively, were treated with corticosteroids before initiating nintedanib treatment. One patient (2.2%) in the non-elderly IPF patient group was treated with cyclosporine in combination with corticosteroids. One patient (5.3%) and two patients (4.4%) in the elderly IPF and non-elderly IPF groups, respectively, who previously received pirfenidone, were switched to nintedanib. One patient (2.2%) in the non-elderly IPF patient group previously received pirfenidone and was administered nintedanib in combination with pirfenidone. There was a significant difference between the two groups in terms of long-term oxygen therapy (LTOT); 14 (73.7%) elderly IPF patients and 17 (37.0%) non-elderly IPF patients were treated with LTOT (*p* = 0.007) at baseline. There were no significant differences between both groups in terms of the prevalence of lung cancer, pulmonary hypertension, and acute exacerbation before nintedanib initiation.

**Table 1:** Baseline characteristics of the 65 IPF patients.

	Total	elderly IPF	non-elderly IPF	*p* value
	(n = 65)	(n = 19)	(n = 46)	
Age, (years)	69.9 ± 8.3	78.2 ± 2.1	66.5 ± 7.4	**< 0.001**
Male, n (%)	57 (87.7)	16 (84.2)	41 (89.1)	0.683
Never smoker, n (%)	12 (18.5)	4 (21.1)	8 (17.4)	0.735
HRCT findings, n (%)				
UIP	49 (75.4)	17 (89.5)	32 (69.6)	0.119
probable UIP	16 (24.6)	2 (10.5)	14 (30.4)	0.119
Surgical lung biopsy, n (%)	5 (7.7%)	1 (5.3)	4 (8.7)	1.000
BMI, (kg/m^2^)	24.0 ± 3.61	22.6 ± 3.92	24.6 ± 3.35	**0.040**
White blood cell count, (/μL)	7517 ± 2343	7383 ± 2043	7574 ± 2478	0.769
Albumin, (g/dL)	3.95 ± 0.43	3.75 ± 0.54	4.04 ± 0.35	**0.013**
C-reactive protein, (mg/dL)	0.77 ± 1.82	1.48 ± 2.98	0.47 ± 0.90	**0.040**
KL-6, (U/mL) (< 500)	1417 ± 924	1273 ± 1125	1478 ± 823	0.422
Pre-treatment before nintedanib, n (%)				
Corticosteroids	7 (10.8)	1 (5.3)	6 (13.0)	0.663
Immunesuppressant agent	1 (1.5)	0 (0.0)	1 (2.2)	1.000
Antifibrotic agent	4 (6.2)	1 (5.3)	3 (6.5)	1.000
LTOT, n (%)	31 (47.7)	14 (73.7)	17 (37.0)	**0.007**
Comorbidities, n (%)				
Lung cancer	7 (10.8)	2 (10.5)	5 (10.9)	1.000
Pulmonary hypertension	4 (6.2)	1 (5.3)	3 (6.5)	1.000
Acute exacerbation	2 (3.1)	1 (5.3)	1 (2.2)	0.502
modified MRC (0 / 1 / 2 / 3 / 4), n	1 / 12 / 26 / 23 / 3	1 / 1 / 4 / 12 / 1	0 / 11 / 22 / 11 / 2	**0.012**
GAP stage (I / II / III), n	10 / 36 / 19	1 / 9 / 9	9 / 27 / 10	**0.025**
Pulmonary function test				
FVC, (mL)	2,393 ± 695	2,052 ± 683	2,534 ± 656	**0.010**
%FVC, (%)	70.6 ± 16.8	67.6 ± 18.3	71.9 ± 16.2	0.347
%DLco (%)	36.9 ± 15.1	25.7 ± 11.7	41.0 ± 14.1	**< 0.001**

Data have been presented as mean (SD), or number (%).

Abbreviations: BMI: body mass index, DLco: diffusing capacity of the lung for carbon monoxide, FVC: forced vital capacity, HRCT: high-resolution computed tomography, IPF: idiopathic pulmonary fibrosis, KL-6: Krebs von den Lungen-6, LTOT: long-term oxygen therapy, MRC: Medical Research Council, UIP: usual interstitial pneumonia

The modified Medical Research Council Dyspnea scale at baseline was more severe in the elderly IPF patient group than in the non-elderly IPF patient group (*p* = 0.012). The GAP stage was significantly more severe in the elderly IPF patient group (Stages I/II/III: 1 [5.3%], 9 [47.4%], and 9 [47.4%], respectively), than in the non-elderly IPF (Stages I/II/III: 9 [19.6%], 27 [58.7%], and 10 [21.7%], respectively) patient group (*p* = 0.025). The respective baseline pulmonary functions of the elderly IPF and non-elderly IPF groups were as follows: FVC (2,052 ± 683 mL vs. 2,534 ± 656 mL; *p* = 0.010), %FVC (67.6 ± 18.3% vs. 71.9 ± 16.2%; *p* = 0.347), and percent predicted DLco (%DLco) (25.7 ± 11.7% vs. 41.0 ± 14.1%; *p* < 0.001). FVC and %DLco were significantly lower in elderly IPF patients. Four elderly IPF patients and three non-elderly IPF patients could not undergo the measurement of DLco at baseline due to disease progression.

### 3.2 Tolerability of nintedanib

The median duration with nintedanib treatment was 350 (range; 13–1573) days (Table 2). Continuation rate of nintedanib treatment at 6 months and at 1 year was not significantly different between the two groups (*p* = 0.289 and 0.174 respectively).

**Table 2:** Nintedanib treatment.

	Total	elderly IPF	non-elderly IPF	*p* value
	(n = 65)	(n = 19)	(n = 46)	
Duration with nintedanib treatment, days (range)	350 (13–1573)	212 (14–1278)	367 (13–1573)	0.050
Continuation rate at 6 months, n (%)	47 (73.3)	12 (63.2)	35 (76.1)	0.289
Continuation rate rate at 1 year, n (%)	29 (44.6)	6 (31.6)	23 (50.0)	0.174
Dose reduction, n (%)	32 (49.2)	9 (47.4)	23 (50.0)	0.847
Duration of dose reduction, days (range)	105 (3–1026)	17 (3–344)	173 (10–1026)	**0.049**
Drug discontinuation, n (%)	27 (41.5)	11 (57.9)	16 (34.8)	0.085
Duration of discontinuation, days (range)	176 (13–1353)	77 (14–630)	191 (13–1353)	0.311
Adverse events associated nintedanib, n (%)	15 (23.1)	6 (31.6)	9 (19.6)	0.340
Progression of IPF, n (%)	4 (6.2)	3 (15.8)	1 (2.2)	**0.038**
Death, n (%)	7 (10.8)	2 (10.5)	5 (10.9)	0.670
Lung transplantation, n (%)	1 (1.5)	0 (0.0)	1 (2.2)	0.517

Data have been presented as median (range), or number (%).

Overall, in this study, dose reductions of nintedanib were observed in 32 (49.2%) patients with no significant difference between both groups; nine (47.4%) in the elderly IPF and 23 (50.0%) in non-elderly IPF groups (*p* = 0.847). Conversely, the duration of dose reduction in the elderly IPF group was significantly shorter than that in the non-elderly IPF group (17 [range, 3–344] days, 173 [range, 10–1026] days, respectively, *p* = 0.049).

A total of 27 (23.1%) patients discontinued nintedanib during the follow-up period. The common reasons for drug discontinuation were adverse events experienced, progression of IPF, and death. One patient discontinued nintedanib owing to lung transplantation. Furthermore, six (31.6%) elderly IPF and nine (19.6%) non-elderly IPF patients discontinued nintedanib because of adverse events. This was not statistically significant (*p* = 0.340).

### 3.3 Adverse events of nintedanib

Adverse events with nintedanib are shown in Table 3. Adverse events occurred in 51 (78.5%) patients; 18 (94.7%) elderly IPF patients and 33 (71.7%) non-elderly IPF patients (*p* = 0.049). The most frequent adverse event was diarrhea, which was observed in 10 (52.6%) elderly IPF patients and 26 (56.5%) non-elderly IPF patients, with no significant difference (p = 0.774). Diarrhea grades 3 or more was experienced by only one elderly IPF patient. Probiotic or loperamide was mostly used as antidiarrheal agents. The second-most frequent adverse event was nausea. Although the frequency of nausea in the elderly IPF group was higher than that in the non-elderly IPF group (10 [52.6%] vs. 14 [30.4%]), it was not statistically significant (*p* = 0.092). Mucosal protective agents, such as rebamipide, proton-pump inhibitors, or antiemetic agents (such as domperidone or metoclopramide), were used to treat nausea. In the elderly IPF group, these drugs were used less frequently, although there was no statistical difference. Hepatic enzyme elevation was observed in 15 (23.1%) patients in total, with no significant difference between both groups; four (21.1%) in the elderly IPF and 11 (23.9%) in non-elderly IPF groups (*p* = 1.000).

**Table 3:** Adverse events of nintedanib treatment.

	Total	elderly IPF	non-elderly IPF	*p* value
	(n = 65)	(n = 19)	(n = 46)	
Any adverse events, n (%)	51 (78.5)	18 (94.7)	33 (71.7)	**0.049**
Diarrhea, n (%)	36 (55.4)	10 (52.6)	26 (56.5)	0.774
Grade 1+2 / 3+4+5, n	35 / 1	9 / 1	26 / 0	
Antidiarrheal agent (probiotic bacterium), n (%)	27 (75.0)	7 (70.0)	20 (76.9)	
Antidiarrheal agent (loperamide), n (%)	17 (47.2)	3 (30.0)	14 (53.8)	
Nausea, n (%)	24 (36.9)	10 (52.6)	14 (30.4)	0.092
Grade 1+2 / 3+4+5, n	24 / 0	10 / 0	14 / 0	
Mucosal-protective agent (rebamipide), n (%)	6 (26.1)	1 (10.0)	5 (35.7)	
Proton-pump inhibitor, n (%)	9 (39.1)	2 (20.0)	7 (50.0)	
Antiemetic agent (domperidon, metoclopramide), n (%)	4 (17.4)	0 (0.0)	4 (28.6)	
Hepatic enzyme elevation, n (%)	15 (23.1)	4 (21.1)	11 (23.9)	1.000
Grade 1+2 / 3+4+5, n	12 / 3	3 / 1	9 / 2	
Thrombocytopenia, n (%)	2 (3.1)	1 (5.2)	1 (2.2)	0.502
Grade 1+2 / 3+4+5, n	2 / 0	1 / 0	1 / 0	

Data have been presented as median (range), or number (%).

Abbreviations: AE: adverse event, IPF: idiopathic pulmonary fibrosis.

The risk factors for any adverse events were evaluated, and GAP stage, serum albumin, and BMI were selected as candidate risk factors. Multivariate logistic analysis revealed an association between BMI and adverse events (Table 4).

**Table 4:** Multivariate logistic regression analysis for any adverse events.

	Odds ratio	95% CIs	*p* value
GAP stage I+II or III	1.595	0.266–9.568	0.609
BMI	0.779	0.622–0.974	**0.028**
serum albumin	1.091	0.212–5.624	0.917

Abbreviations: BMI: body mass index, CI: confidence interval.

### 3.4 Efficacy of nintedanib

**Fig 1:**
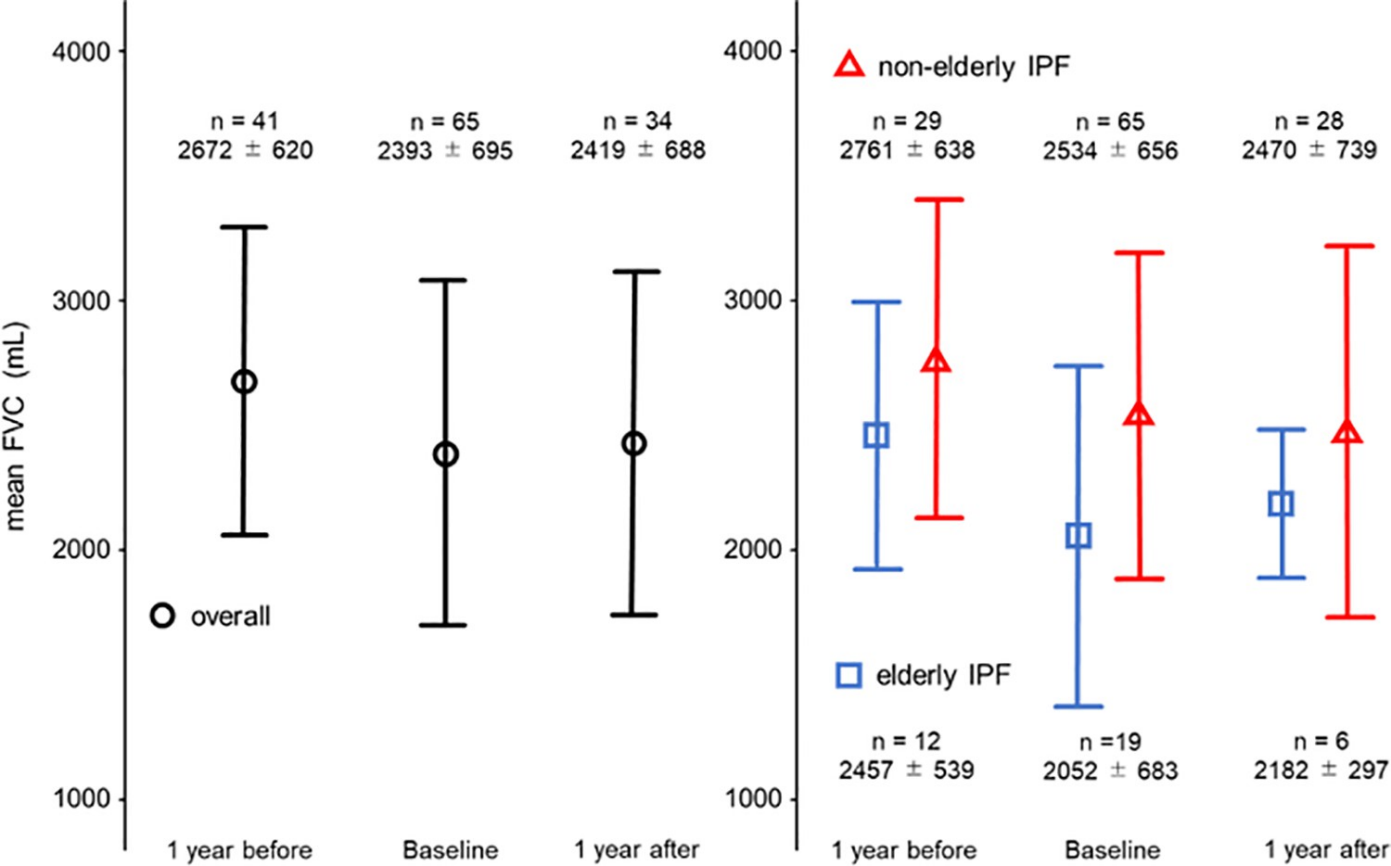
The mean FVC of IPF patients treated with nintedanib. Changes in mean FVC (±SD) at 1 year before, at baseline, and at 1 year after nintedanib initiation for IPF patients. There was a reduction of FVC decline after nintedanib initiation in all groups. (Black: overall group, blue: elderly IPF group, red: non-elderly IPF group). Abbreviations: FVC: forced vital capacity, IPF: idiopathic pulmonary fibrosis, SD: standard deviation.

FVC changes at 1 year before, baseline, and 1 year after nintedanib initiation are shown in Fig 1. The mean FVC at 1 year prior to nintedanib initiation was 2457 ± 539 mL and 2761 ± 638 mL in the elderly IPF and non-elderly IPF groups, respectively. The mean FVC at 1 year after nintedanib initiation was 2182 ± 297 mL and 2470 ± 739 mL in the elderly IPF and non-elderly IPF groups, respectively.

**Fig 2:**
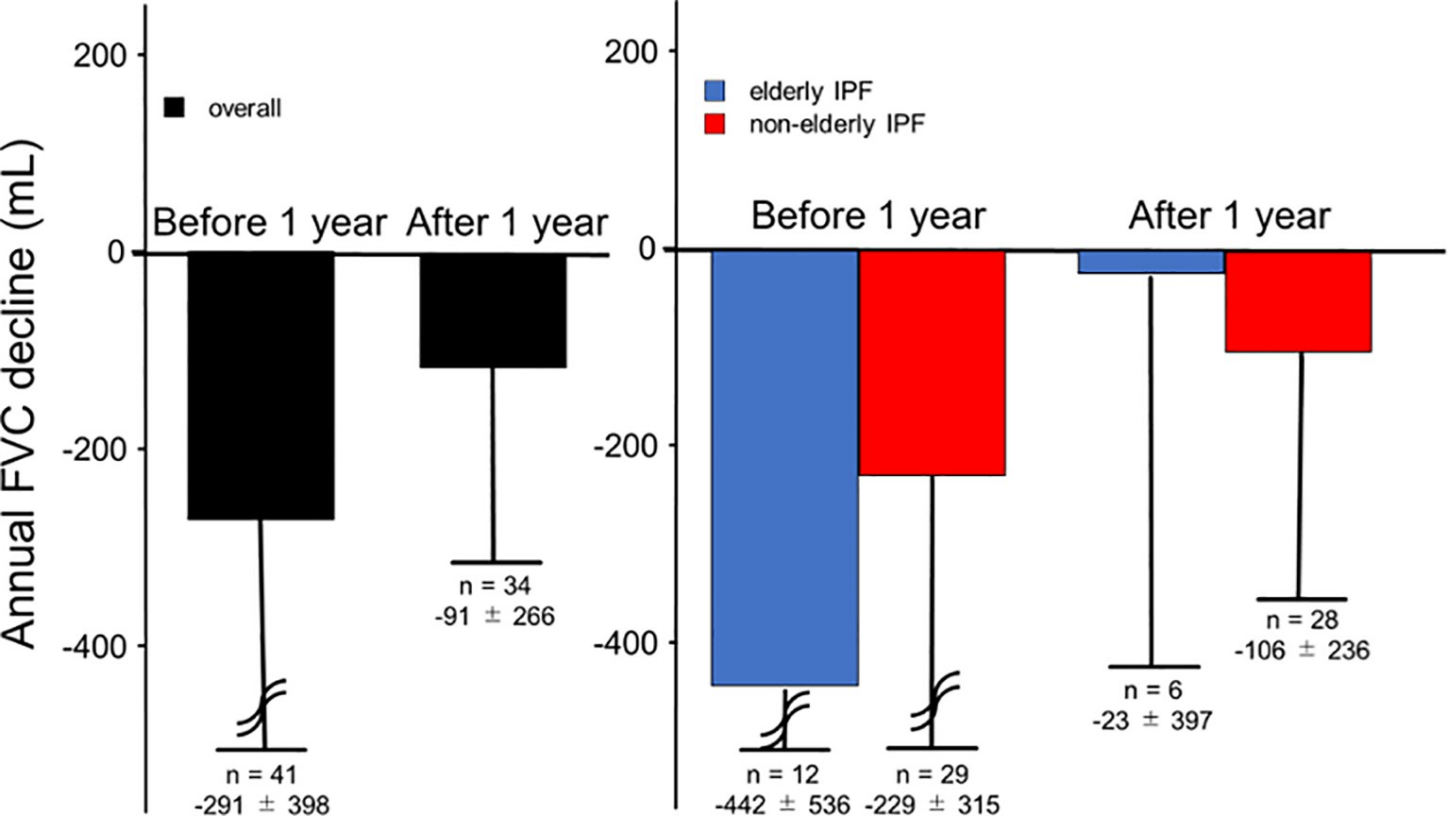
Annual FVC decline before and after nintedanib initiation. Changes in annual FVC decline (mean ± SD) 1 year before and after nintedanib initiation for IPF. There was a similar reduction of annual FVC decline in both the elderly and non-elderly groups. (Black: overall group, blue: elderly IPF group, red: non-elderly IPF group). Abbreviations: FVC: forced vital capacity, IPF: idiopathic pulmonary fibrosis, SD: standard deviation.

Annual FVC decline in 1 year before and after nintedanib initiation are shown in Fig 2. FVC decline 1 year before and after nintedanib initiation was -291 ± 398 mL and -91 ± 266 mL among all patients. After nintedanib initiation, annual FVC decline was reduced, though there was no statistical difference (*p* = 0.162). FVC decline 1 year before and after nintedanib initiation was -442 ± 536 mL and -23 ± 397 mL in the elderly IPF group and -229 ± 315 and -106 ± 236 mL in the non-elderly IPF group. Although the annual FVC decline decreased after nintedanib initiation in both groups, there was no statistical significance (*p* = 0.375 and 0.561, respectively).

The median follow-up period was 449 (range; 29–1573) days (Table 5). During the follow-up period, lung cancer developed in two (10.5%) elderly IPF and two (4.3%) non-elderly IPF patients. Acute exacerbation developed in six (31.6%) elderly and 11 (23.9%) non-elderly IPF patients after nintedanib initiation. Four elderly IPF patients died of acute exacerbation, three of chronic respiratory failure, and one of lung cancer. In contrast, four non-elderly IPF patients died of acute exacerbation, four of chronic respiratory failure, one of lung cancer, and one of cardiac failure.

**Table 5:** Comorbidities and prognoses of IPF patients after nintedanib initiation.

			Total	elderly IPF	non-elderly IPF
			(n = 65)	(n = 19)	(n = 46)
Follow-up period, day, (range)			449 (29–1573)	391 (29–1278)	560 (76–1573)
Comorbidities after nintedanib initiation, n (%)					
Lung cancer			4 (6.2)	2 (10.5)	2 (4.3)
Acute exacerbation			17 (26.2)	6 (31.6)	11 (23.9)
Death, n (%)			19 (29.2)	8 (42.1)	11 (23.9)
Acute exacerbation			8 (42.1)	4 (50.0)	4 (36.4)
Chronic respiratory failure			7 (36.8)	3 (37.5)	4 (36.4)
Lung cancer			2 (10.5)	1 (12.5)	1 (9.1)
Cardiac failure			1 (5.3)	0 (0.0)	1 (9.1)
other			1 (5.3)	0 (0.0)	1 (9.1)

Abbreviations: IPF: idiopathic pulmonary fibrosis.

**Fig 3:**
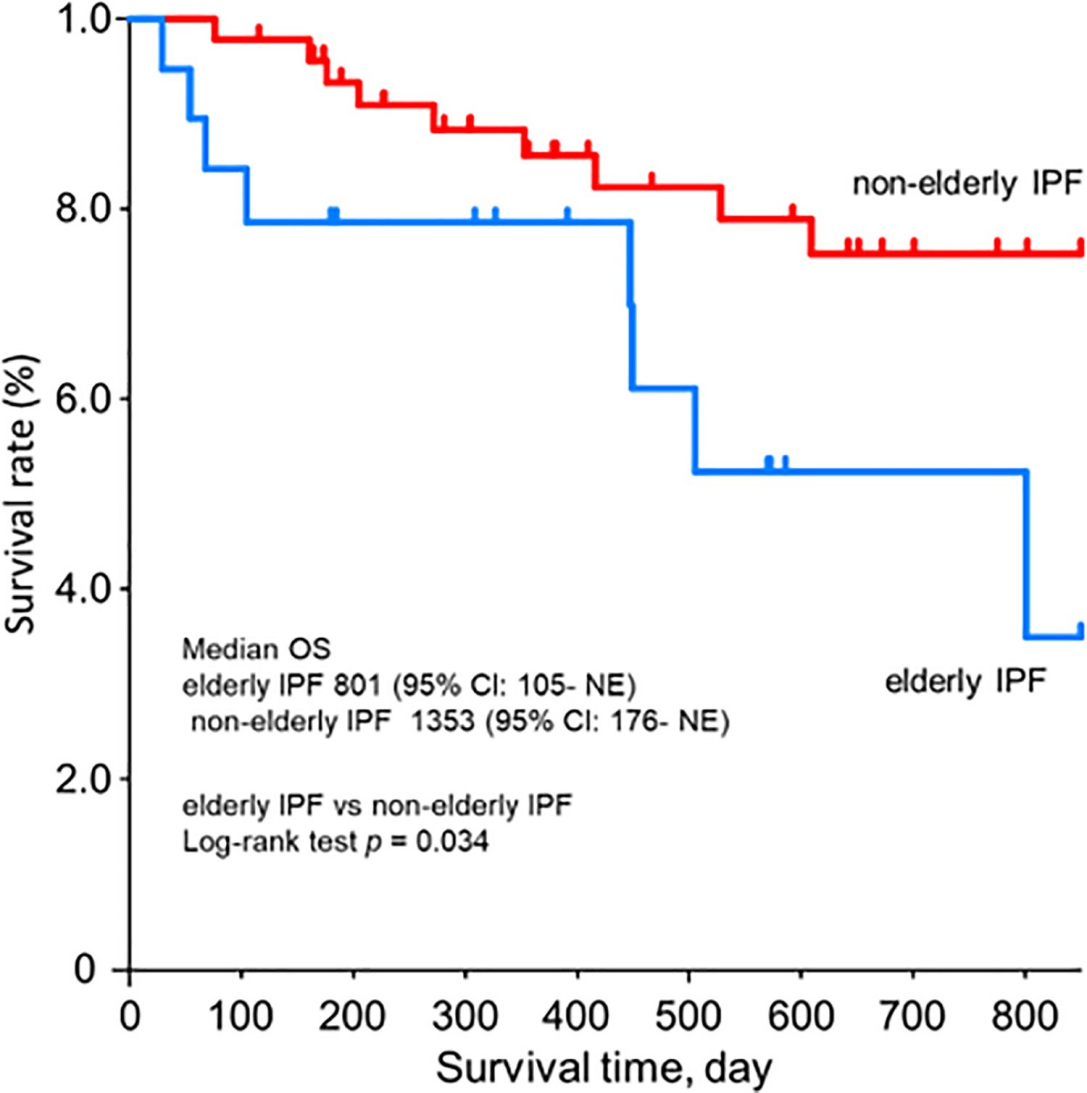
Survival curves of elderly IPF and non-elderly IPF after nintedanib initiation. The Kaplan-Meier curve of the overall survival of the IPF patients (elderly IPF, and non-elderly IPF). (Blue: elderly IPF, red: non-elderly IPF). Abbreviations: CIs: confidence intervals, IPF: idiopathic pulmonary fibrosis, NE: not evaluable, OS: overall survival.

The Kaplan–Meier curves of the OS from nintedanib initiation are shown in Fig 3. There were statistical differences between the two groups, elderly and non-elderly IPF (*p* = 0.034); the prognosis of elderly IPF was worse than non-elderly IPF.

## 4. Discussion

This study revealed that the elderly IPF group experienced more adverse events of nintedanib, despite the discontinuation rate of the drug due to adverse events being equivalent in both patient groups. The Duration of dose reduction of nintedanib in the elderly IPF group was shorter than that in the non-elderly IPF group. On the other hand, the similar continuation rates at 6 months and 1 year demonstrates similar tolerability. These results suggest that long-term therapy with nintedanib in elderly IPF patients may be possible if there is proper management for adverse events, including dose reduction. Consequently, clinicians should not avoid treating patients with nintedanib based solely on their age.

In this study, there were 19 (29.2%) elderly IPF patients. It is well known that IPF is more prevalent in the elderly, and its incidence increases with age [1, 17, 21]. In our study, physiological variables such as FVC and DLco of elderly IPF patients were lower than those of the non-elderly IPF patients. The decreased pulmonary function and age might contribute to the level of dyspnea and disease severity among elderly IPF patients. Generally, the poorer the health status of patients, the higher is the risk of drug-related adverse events. In fact, many physicians hesitate to initiate antifibrotic agents because of their concern for its adverse event profile [22]. Although some reports have demonstrated the efficacy of nintedanib in early-stage IPF [23–25], many patients are only diagnosed with the disease at an advanced age. Therefore, this presents a dilemma whether to treat elderly IPF patients with an antifibrotic agent or only with palliative therapy [17].

The tolerability of nintedanib among elderly IPF patients was demonstrated by the continuation rates at 6 months and 1-year post-initiation. There was no difference in the severity of adverse events in both patient groups. There was also no difference between the two groups with respect to supportive therapy, such as antidiarrheal agents or anti-nausea agents. However, these two groups differed in duration of dose reduction with nintedanib. It was presumed that in the elderly IPF patients, the dose reduction was made at an early stage of the mild adverse events. This indicates that dose reduction of nintedanib during an early stage may be necessary, even with mild adverse events, for elderly IPF patients. Appropriate pharmaceutical care is recommended for IPF patients on chronic nintedanib therapy, including dose titration and temporary drug discontinuation.

The elderly IPF patient group had a more severe state of disease compared to the non-elderly IPF patient group. Abe et al. reported that severe IPF patients experienced more adverse events of nintedanib than did the mild to moderate group [26]. In patients with severe IPF, the continuation of nintedanib treatment for more than 3 months might lead to a better prognosis compared to those who do not continue the treatment [26]. Therefore, the dose of nintedanib should be titrated over time to the maximum recommended dose for elderly IPF patients with low physiological variables.

In addition, the elderly IPF group had a low serum albumin level compared with that of the non-elderly group. This might correlate to a higher frequency of adverse events in the elderly IPF patient group. Serum albumin level affects a drug’s pharmacokinetics. Although there was no significant difference between serum albumin level and adverse events in this present study, caution should be exercised when initiating nintedanib in elderly IPF patients with low serum albumin levels.

It was found that patients with a low BMI had a higher risk of an adverse event. A previous study reported that a low BMI was a significant risk factor for diarrhea [27]. Therefore, when initiating nintedanib treatment in IPF patients, those with low BMI scores should be carefully managed irrespective of whether the patients are elderly or not.

In this study, the frequency of nausea was relatively high compared with that in previous randomized trials [11, 15, 16] and similar to those of other real-world studies [19, 27]. Nausea may be induced not only by nintedanib but also by other factors. Proesmans et al. reported that nausea occurred in 20% of IPF patients with non-drug treatment [28]. Disease progression itself might induce nausea. In elderly IPF patients, reduced gastrointestinal and metabolic function associated with aging, as well as irregular mealtimes, may be associated with nausea [29]. The management of nausea is important as it may deter patients from continuing pharmacotherapy, which could worsen the disease. Furthermore, Kato et al. reported that poor health status and low BMI were associated with nausea during nintedanib treatment [27]. In this study, although the nausea was all Grade 1+2, careful consideration was necessary, such as dose titration and temporary discontinuation of nintedanib treatment in elderly IPF patients. When nausea persists, it is recommended that medication be taken together with food [28].

The CRP level was higher in elderly IPF patients than in non-elderly IPF patients; however, the cause for this difference was unclear. Although other causes of ILD, such as collagen disease, have been excluded with MDD, it cannot be ruled out that diseases other than IPF may be included due to the inclusion of a few cases with surgical lung biopsy samples. Additionally, elderly IPF patients might have been more infectious than non-elderly IPF patients due to aging, low levels of albumin, and low BMI.

Our study demonstrated a statistically non-significant reduction in annual FVC decline before and after nintedanib initiation. The result could be explained as a limitation of the study in that all patients could not perform follow-up pulmonary function tests because of disease progression, hospital transfer, or death. Additional post-hoc analysis of INPULSIS trials indicated that the efficacy of nintedanib was not affected by age (< 65, or ≥ 65 years old) [23]. Takeda et al. reported that nintedanib also improves subjective improvement, as well as FVC decline in elderly IPF patients [18]. These studies suggest that nintedanib is effective for elderly IPF patients.

Our study had some limitations. First, this was a single-center study with a small sample size. Studies with larger sample sizes are needed to validate our findings. Additionally, there was an unavoidable selection bias since not all elderly IPF patients were treated with nintedanib. Thus, it is undeniable that the patients participating in this study might have been in good condition. Second, there were several patients who did not undergo follow-up pulmonary function tests. This prevented a conclusive evaluation of the annual FVC decline. A prospective study design with well-defined endpoints is therefore advocated. Third, as this study evaluated nintedanib over a short period, a longer-term study to evaluate the tolerability of the drug is required.

## 5. Conclusions

Nintedanib treatment was tolerable in elderly IPF patients and appropriate in the therapeutic management of adverse events. In addition, elderly IPF patients could use nintedanib as chronic therapy. Careful patient management may be necessary when initiating treatment with nintedanib in elderly IPF patients.

## Supporting information

S1 Data (XLSX)

S2 Data (XLSX)
